# Investigating risk factors for under-five mortality in an HIV hyper-endemic area of rural South Africa, from 2000-2014

**DOI:** 10.1371/journal.pone.0207294

**Published:** 2018-11-26

**Authors:** B. Tlou, B. Sartorius, F. Tanser

**Affiliations:** 1 Discipline of Public Health Medicine, School of Nursing and Public Health, University of KwaZulu-Natal, Durban, South Africa; 2 Africa Health Research Institute, KwaZulu-Natal, South Africa; 3 Centre for the AIDS Programme of Research in South Africa—CAPRISA, University of KwaZulu-Natal, Congella, South Africa; 4 Research Department of Infection and Population Health, University College London, London, United Kingdom; University of Cape Town, SOUTH AFRICA

## Abstract

**Introduction:**

Despite global progress, there remains a disproportionate burden of under-five year old deaths in sub-Saharan Africa (SSA), where four out of five child deaths occur. Substantial progress has been made in improving sanitation, controlling communicable diseases and the spread of HIV in most parts of the world. However, significant strides to address some key risk factors related to under-five mortality are still needed in rural SSA if they are to attain relevant 2030 SDG targets. The aim of this study is to investigate the risk factors for under-five mortality in an HIV hyper-endemic area of rural South Africa, from 2000–2014. Some of the key risk factors investigated are, for example: household wealth, source of drinking water, distance to the national road and birth order.

**Methods:**

We conducted a statistical analysis of 759 births from a population-based cohort in rural KwaZulu-Natal Province, South Africa, from 2000 to 2014. A Cox Proportional Hazards model was used to identify the risk factors and key socio-demographic correlates of under-five mortality leveraging the longitudinal structure of the population cohort.

**Results:**

Child mortality rates declined by 80 per cent from 2000 to 2014, from >140 per 1,000 persons in years 2001–2003 to 20 per 1,000 persons in the year 2014. The highest under-five mortality rate was recorded in 2002/2003, which decreased following the start of antiretroviral therapy rollout in 2003/4. The results indicated that under-five and infant mortality are significantly associated with a low wealth index of 1.49 (1.007–2.48) for under-fives and 3.03 (1.72–5.34) for infants. Children and infants with a lower wealth index had a significantly increased risk of mortality as compared to those with a high wealth index. Other significant factors included: source of household drinking water (borehole) 3.03 (1.72–5.34) for under-fives and 2.98 (1.62–5.49) for infants; having an HIV positive mother 4.22 (2.68–6.65) for under-fives and 3.26 (1.93–5.51) for infants, and period of death 9.13 (5.70–14.6) for under-fives and 1.28 (0.75–2.20) for infants. Wealth index had the largest population attributable fraction of 25.4 per cent.

**Conclusions:**

The research findings show a substantial overall reduction in under-five mortality since 2003. Unsafe household water sources and having an HIV-positive mother were associated with an increased risk of under-five mortality in this rural setting. The significant risk factors identified align well with the SDG 2030 targets for reducing child mortality, which include improved nutrition, sanitation, hygiene and reduced HIV infections. Current trajectories suggest that there is some hope for meeting the 2030 SGD targets in rural South Africa and the region if the identified significant risk factors are adequately addressed.

## Introduction

There has been an impressive global improvement in child deaths reduction since 1990, with statistics showing that under-five deaths decreased from 12.7 to 5.9 million from 1990 to 2015. In addition, approximately 16,000 children die daily compared to about 35,000 in 1990 [1]. While SSA has a high under-five mortality rate (U5MR), this has decreased considerably in recent years [1]. In South Africa (SA), statistics have indicated an annual child mortality reduction rate of 1.6 per cent, from 60 per 1,000 live births in 1990 to 41 per 1,000 in 2015 [2], despite which, it failed to achieve the Millennium Development Goal 4 (MDG4). The main key drivers of under-five mortality reduction at a global level are maternal education, improvements in public health innovations and rising income. Research has shown that an increase in maternal education and health innovations and policies as well as rising per capita income has led to under-five mortality reductions globally [2].The United Nations’ new Sustainable Development Goals aim to lower U5MR to as little as 25 per 1,000 live births in all countries by 2030.

The five leading causes of under-five deaths in South Africa are HIV, malnutrition, diarrhea, lower respiratory infections and low weight at birth [3]. Some of the determinants of health that promote these direct causes include socio-economic status, the mother’s education and HIV status, and the source of drinking water [4]. Children born into poverty (less than $1.25 a day) are two times more likely to demise before they reach the age of five compared to those from affluent households [5]. In addition, children born from educated mothers have a high probability of survival compared to those born from uneducated mothers [6].

Despite the decline in childhood mortality, rural areas in South Africa? continue to experience high childhood mortality rates [7], with HIV being the main cause of deaths in children younger than five, responsible for approximately 40% of the cases [2]. This could be directly attributed to mother-to-child transmission of HIV, and indirectly due to high maternal mortality due to HIV [8], and also the absence of mothers may imply a lack of care for the children. Only about 32 per cent of the approximately 2.6 million children living with HIV have been diagnosed, and less than 32 per cent of these have had access to ART since 2014 [9]. While most of these deaths are preventable, the lack of transparent health intervention programmes and appropriate policies have exacerbated the already high U5MR in SSA [10].

Researchers have largely focused on the influence of individual factors on U5MR, with little being known about how community, familial and household characteristics, affect child mortality [11]. Studies in low- and middle-income nations have indicated that household composition plays a central role in child mortality, with few longitudinal studies having examined this influence in HIV endemic areas [12, 13, 14]. This also applies in rural South Africa, where little attention has been paid to contextual risk factors, such as household composition and socio-economic status, as factors of U5MR [14] and the extent to which they contribute to these deaths.

It is hoped that this investigation will enable the responsible authorities to develop global health policies and assess the potential population effects of significant risk factors [5], particularly in high mortality and HIV hyper-endemic settings such as the study area. The results will also be presented from a broad analysis of child mortality risk factors, and their varying effects by age, over a period of 14 years in a rural area of South Africa. This could help the South African Government, non-governmental organisations and other partners in the health sector to target important contextual risk factors in a high HIV endemic setting to attain relevant SDG targets. This study will also estimate the population attributable fraction (PAF) for the identified risk factors for under-five mortality in different age ranges of the first 59 months of life (neonatal, 0–28 days; infant, 1–11 months; and under-5, 0–59 months).

## Methods

### Study design

This is a population-based cohort study conducted using data obtained from the Africa Centre Demographic Information System (ACDIS) in South Africa from 2000–2014, the longitudinal observations being conducted on individuals, households and physical structures (clinics, schools and homesteads). Data was collected via questionnaires administered to key household informants.

### Study area

**Fig 1:**
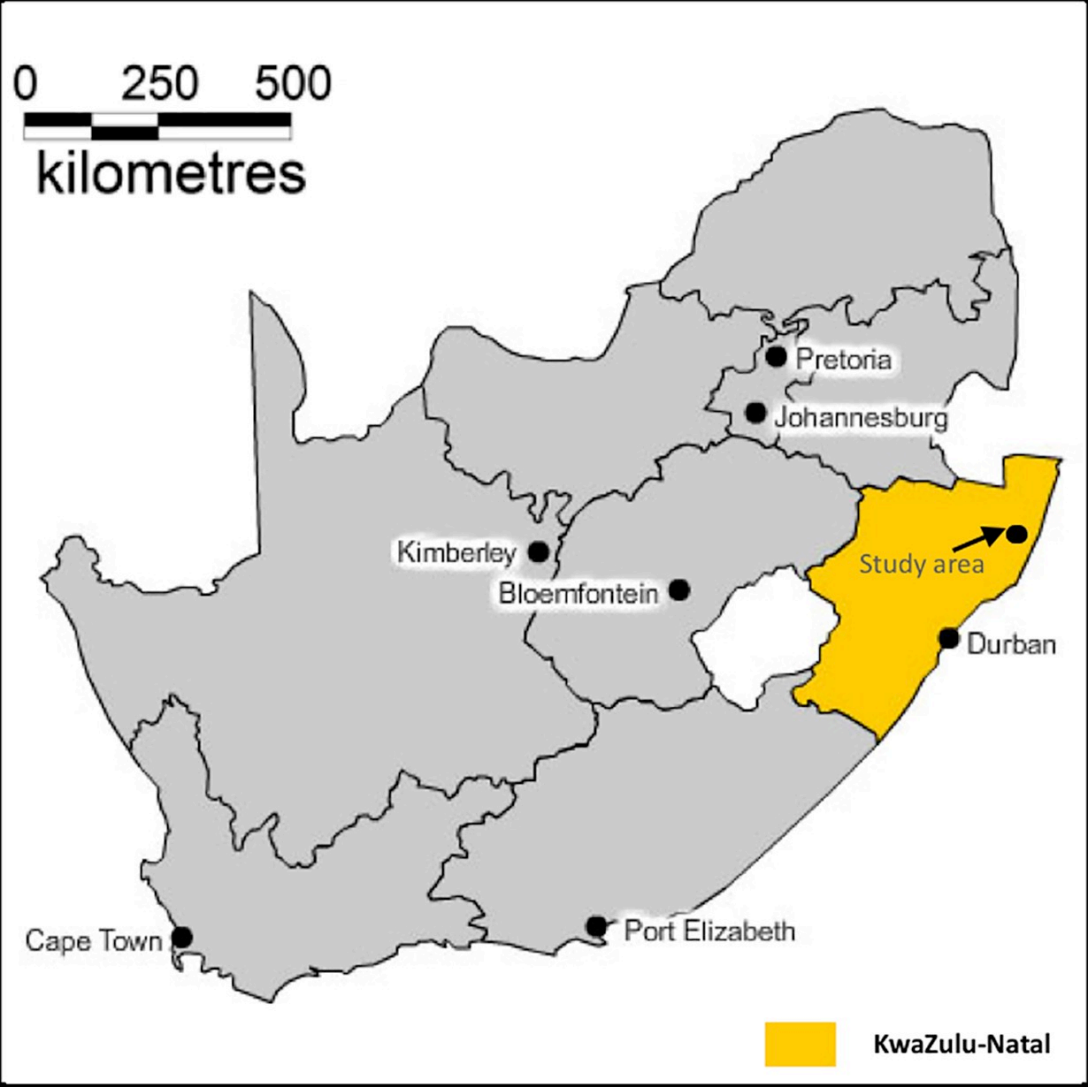
Location of the Africa Centre demographic surveillance site in KwaZulu-Natal, South Africa.

The Africa Centre (now Africa Health Research Institute) Demographic Information System (Fig 1) is located in a rural sub-district of uMkhanyakude in northern KwaZulu-Natal Province, South Africa [15]. The Africa Centre Demographic Information System has been in existence since 2000, and contains a detailed demographic profile of a rural population of approximately 90,000 individuals from 11,000 households (approximately 7.9 members per household), across an area of 438 km [15].

The demographic surveillance system dynamically maintains demographic events (births, deaths, migrations, marriages, relationships) of all people residing within a geographically defined area [16], with follow-up being carried out two (until 2012) or three (since 2012) times annually through routine household visits done by trained field workers, with an estimated surveillance participation rate greater than 99 per cent [15]. Most of the area’s households are scattered multigenerational homesteads of different sizes [17]. There is a high prevalence of HIV, unemployment and poverty amongst the adults, with the majority of people being poor (two in five are formally employed), while household access to safe water increased rapidly from 42% in 2001 to 80% by 2007 [18, 19]. Most people in the area survive through governmental grants, pensions and waged employment [15].

Teams of trained field workers also visit eligible individuals in their households annually to conduct HIV sero-surveys, while a rapid rollout of antiretroviral therapy (ART) began in 2004 through public sector programmes led by trained nurses, with approximately 67% of patients being female. Recent results from population viral load surveys in this area show that the viral suppression level among females has increased from 28.2% in 2011 to 44.7% in 2014 [20], the ART initiation having accelerated the prevention of mother-to-child transmission programme.

### Study population

The study carried out a risk factor analysis of children less than five years of age who were born, lived or in-migrated as residents and were registered in the surveillance area between 2000 and 2014. The longitudinal platform was utilised in a time-to-event approach, the analysis covering all deaths of children under the age of five years, and details regarding data used in this study, having been reported elsewhere [15, 21, 22, 23, 24].

### Study outcome variables

The main outcome variables in this study were: under-five mortality, defined as death between birth and 59 months; post-neonatal mortality is defined as the infant deaths greater than 27 days but less one year, and a child is defined as a person under five years of age.

### Key risk factors

The potential explanatory variables for this study are based on using the Mosley and Chen framework [11] for determinants of child mortality in third world countries and the relevant literature from similar previous studies [25, 26] conducted on under-five mortality in rural settings.

The predictor variables utilized in this study are mother’s education, mother’s vital status’, HIV status and age, as well as wealth index, source of drinking water, birth order, child sex, distance to the nearest clinic and main road, and the period of death. After written informed consent, HIV status was determined by trained field workers collecting blood by finger pricking and performing the antibody testing according to the World Health Organization (WHO) guidelines. Wealth index is defined as the measurement of varying socio-economic statuses based on asset-based indices that are computed from different households’ assets and quintiles using principal components analysis (PCA). The time periods of 2000–2005 and 2006–2014 are defined as the period before the ART roll-out and the time from the inception of the ART programme in ACDIS respectively. Status is defined as the situation or standing of the variable of interest at a particular time during a process, and period of death is defined as the time from birth until the event of interest or end of the study period. All data were fully anonymised before they were uploaded onto the AHRI repository and this study was approved by the Biomedical Research Ethics Committee (BREC) of the University of KwaZulu-Natal (BE 169/15).

### Statistical analysis

The Cox proportional hazards regression model was used to investigate significant risk factors for under-five mortality in this study area from 2000–2014. The authors observed time (t) for each respondent as the time from birth until death or the date of the fifth birthday, depending on which came first. The observation time for emigrated children was defined as the period from birth until the date of emigration, while for in-migrations, this was the time from in-migration until the date of event of interest. We first assessed relevant risk factors or covariates (e.g. wealth index, water source, HIV status, distance to the national road, birth order and period of death) using univariate Cox regressions. All variables found to be statistically significant in the univariate analysis were then selected for entry into the multivariable model. Any p-value less than 0.05 was deemed statistically significant, and multicollinearity was established between risk factors using Variance Inflation Factors (VIF) [27]. The supremum test was used for proportional hazards to establish whether the proportional hazards assumption was upheld.

We also calculated Population Attributable Fractions or PAFs (in this case the proportion of under five mortality attributable to a given exposure) for significant risk factors using the formula below [28]:

$$PAF=\frac{P_{e}\left(RR-1\right)}{1+P_{e}\left(RR-1\right)}$$

where *P*_*e*_ is the prevalence of exposure in the population and RR is the multivariable adjusted relative risk. PAFs help indicate the number (or proportion) of child deaths that would not occur in a population if a given risk factor were eliminated (e.g. how many lives would be saved if the socio-economic status improved) and thus inform targeting of the most highly attributable risk factors in a given context. The SAS procedure was used for proportional hazards regression (PHREG) version 9.4 (SAS Institute, Cary, NC, USA) [29], which allows the analysis of continuous and time-varying covariates and STATA 13 [30].

## Results

### Description of the Study Population

Table 1 presents the individual, household and community level characteristics of children by mortality status. A total of 759 deaths of children below the age of five years occurred between 2000 and 2014 from a total of 12,989 live births: 274 deaths between 0 and 28 days (neonatal mortality); 277 between one month and 11 months (infant mortality); 208 in the child mortality phase (12–59 months), with the overall U5MR (scaled by live births) being 58.4 deaths per 1,000 live births.

**Table 1:** Demographic variables for infant and under-five deaths in DSA, 2000-2014 (*n* = 759).

Variable	Categories of variable	Infant n (%)	Under 5 n (%)
Maternal education	None	4 (1.5)	8 (1.1)
	Primary	35 (12.8)	74 (9.7)
	Secondary or higher	134 (48.2)	396 (52.2)
	Unknown	104 (37.6)	281 (37.0)
Mother’s Vital Status	Dead	5 (1.8)	14 (1.8)
	Alive	272 (98.2)	745 (98.2)
Mother’s HIV Status	Positive	22 (8.1)	64 (8.4)
	Negative	245 (88.3)	599 (78.9)
	Unknown	10 (3.6)	96 (12.7)
Wealth Index	Poor	137 (49.6)	389 (51.2)
	Middle	54 (19.5)	146 (19.2)
	Rich	82 (29.7)	190 (25)
	Unknown	3 (1.2)	35 (4.6)
Source of drinking water	Borehole	62 (22.5)	168 (22.1)
	Well	41 (14.7)	84 (11)
	Surface/rain/lake	35 (12.7)	140 (18.4)
	Water tank	6 (2.0)	22 (2.9)
	Piped water	133 (48.0)	346 (45.6)
Birth order	First	119 (42.9)	291 (38.4)
	2^nd^, 3^rd^ or 4th	124 (44.9)	316 (41.6)
	Fifth or higher	25 (9.0)	58 (7.6)
	Unknown	9 (3.2)	93 (12.3)
Child’s Sex	Male	138 (49.9)	395 (52.1)
	Female	139 (50.1)	364 (47.9)
Mother’s age (years) at birth	<20	33 (11.8)	77 (10.2)
	20–29	142 (51.1)	406 (53.5)
	30–39	57 (20.5)	247 (32.6)
	40 and above	52 (18.6)	28 (3.7)
Distance to national road (km)	<10	180 (65.1)	524 (69)
	≥10	97 (34.9)	235 (31)
Distance to nearest clinic (km)	<10	274 (99.0)	502 (66.1)
	≥10	3 (1.0)	257 (33.9)
Period of death	2000–2005	189 (68.2)	542 (71.4)
	2006–2014	88 (31.8)	217 (28.6)

Approximately 70 per cent of the neonatal, infant, child and under-five deaths occurred between 2000 and 2005, with households in the poor quintile having a higher percentage of deaths than wealthier ones (57.1% neonatal, 50.6% infant, and 52.2% under-5). The frequency of male deaths was slightly higher than that of female deaths but this difference was not statistically significant (52.1% vs 47.9%; p = 0.484).

**Fig 2:**
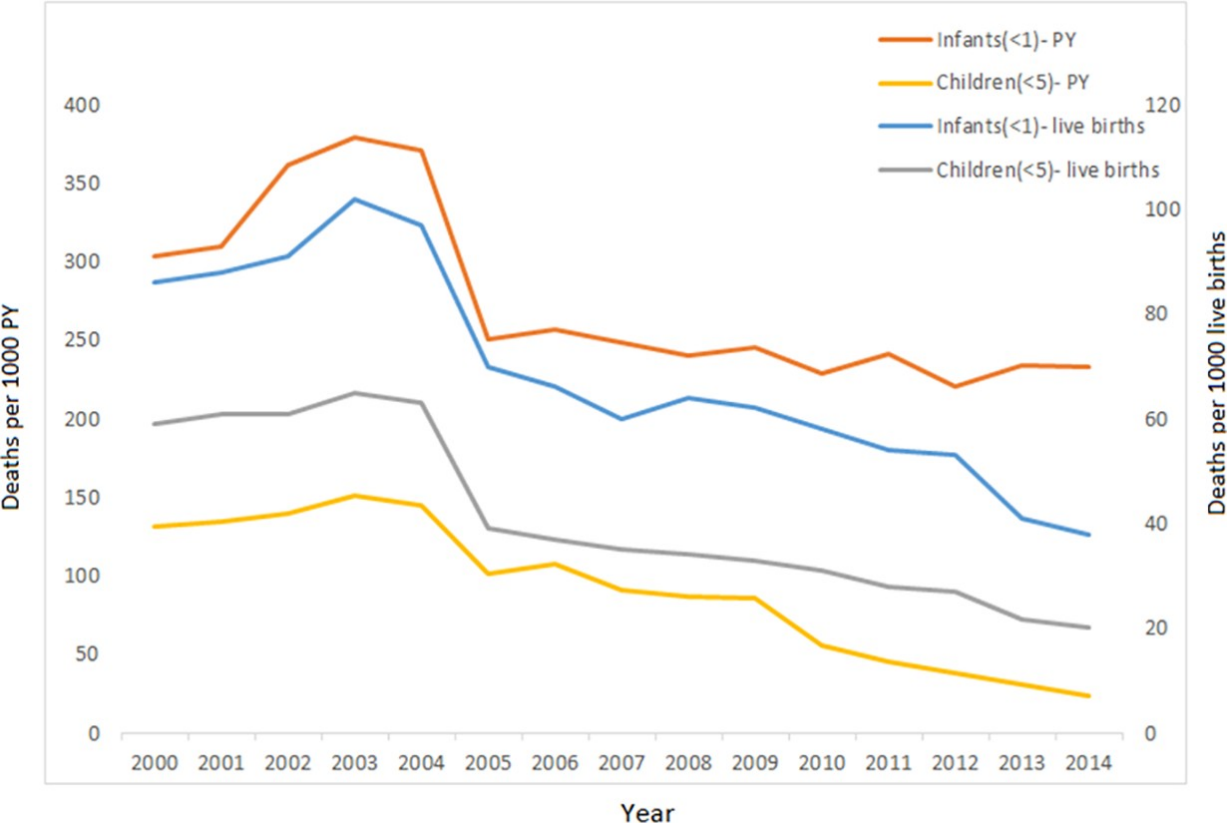
Infant and child deaths per 1,000 live births and person-years between 2000 and 2014.

Mortality rates per 1,000 live births declined in all child age groups from 2000 to 2014. (Table 2). Between 2000–2005 and 2006–2013, the neonatal mortality rate decreased by approximately six per cent, from 767 deaths per 1,000 person years among live born children (2000–2005) to 722 deaths in 2006–2014 even though the decrease was not statistically significant; infant mortality fell from approximately 50 to 20 deaths; post-neonatal mortality declined by 38 per cent from 379 to 234 deaths; the child mortality rate reduced by approximately 40 per cent, from 33 to 20, and the U5MR dropped by 85 per cent, from 149 to 23 deaths, as shown in Fig 2.

**Table 2:** Trends in early childhood mortality rates in deaths per 1,000-person years among live born children.

Characteristic	2000–2005	2006–2014	p-value
**Neonatal mortality (*N*)**	767.2	722.8	0.5029
**Post neonatal mortality (*PN*)**	379	234.8	0.0011
**Infant mortality (1*q*0)**	354.8	215.2	0.0002
**Child mortality (4*q*1)**	33.2	12	<0.001
**Under 5 mortality (5*q*0)**	151	23.1	<0.001

Neonatal mortality (*N*)-refers to the probability of demising within the first month of life

Post neonatal mortality (*PN*)-refers to the difference between neonatal and infant mortality

Infant mortality (1*qo*)-refers to the probability of dying in the first year of life

Child mortality (4*q*1)-refers to the probability of demising between exact age one and five

Under 5 mortality (5*q*0)-refers to the probability of demising between birth and exact age five.

### Risk Factors for Infant Mortality (1–11 months)

Infants born to mothers from poor households were at 50% higher risk (HR = 1.49, CI 1.01 to 2.48) of mortality than those born from richer households (Table 3). In addition, infant livings in households who used boreholes and wells were at a 3 fold higher risk of dying compared to those who drank piped water (HR = 2.98, CI 1.62 to 5.49; HR = 2.98, CI 1.39 to 6.40 respectively). Mother’s HIV status was also significantly associated with infant mortality i.e. infants with HIV positive mothers were greater than 3 fold more likely to die compared to those born to HIV negative mothers (HR = 3.26, CI 1.93 to 5.51).

**Table 3:** Hazard ratios for potential determinants of infant mortality in rural KwaZulu Natal (South Africa) using a Cox proportional hazards regression model.

		Univariable			Multivariable					
Explanatory variables	Categories of explanatory variables	Hazard ratio	95% Confidence Interval	p- value	Adjusted Hazard Ratio	95% Confidence interval	p-value	Relative risk	PAF (%)	95%CI
**Wealth Index**	Poor	2.09	1.35–3.24	0.001	1.49	1.007–2.48	0.047	1.59	25.4	4.4–43.6
	Rich	1			1					
**Source of drinking water**	Borehole	2.72	1.69–4.35	<0.001	2.98	1.62–5.49	<0.001	2.46	15	7.7–23.8
	Well	2.42	1.36–4.29	0.003	2.98	1.39–6.40	<0.001	2.20	8.1	2.2–16.6
	Surface/rain/lake	1.95	1.17–3.24	0.010	1.86	0.89–3.86	0.096	1.54	6.9	-0.8–17.2
	Other (water tank)	0.59	0.21–1.64	0.314	0.80	0.24–2.65	0.719	0.87		
	Piped water	1			1					
**Mother’s HIV Status**	Positive	1.66	1.29–2.13	<0.001	3.26	1.93–5.51	<0.001	2.22	9.3	4.8–15.
	Negative	1			1					
**Distance to national road(km)**	≥10	1.26	1.01–1.57	0.038	1.45	0.90–2.34	0.122	1.34	9.5	-1.6–21.6
	< 10	1			1					
**Birth order**	First	0.76	0.63–0.91	0.003	0.79	0.48–1.30	0.346			
	2^nd^, 3^rd^ or 4th	1			1			1.4	16	-1.9–33.4
	Fifth or bigger	0.99	0.74–1.33	0.943	0.77	0.40–1.50	0.438	1.2	2.1	-3.0–9.9
**Period of death**	2000–2005	1.86	1.54–2.23	<0.001	1.28	0.75–2.20	0.369	1.20	10.6	-11.3–34.6
	2006–2014	1			1					
**Maternal education**	none	0.89	0.44–1.79	0.738						
	Primary	0.91	0.69–1.19	0.480						
	Secondary	1								
**Mother’s Vital Status**	Dead	3.11	1.75–5.52	<0.421						
	Alive	1								
**Mother’s age(years) at birth**	< 20	0.87	0.62–1.21	0.397						
	20–29	1.01	0.81–1.25	0.973						
	30–39	1								
	≥ 40	1.54	0.90–2.65	0.115						
**Child’s Sex**	Male	1.02	0.86–1.21	0.827						
	Female	1								
**Distance to the nearest clinic(km)**	≥10	0.68	0.28–1.63	0.383						
	<10	1								

### Risk Factors for Under-Five Mortality (age 0–59 months)

The results of the univariate and multivariable risk factor analysis for child mortality are shown in Table 4. After multivariable adjustment, lower wealth index, source of drinking water, mother’s HIV status and period of death remained significant risk factors. The results showed that children living in poor socio-economic households had a 2.5-fold higher incidence of death compared to those from the wealthier homes (HR = 2.47, CI 1.56 to 3.93). Children residing in households whose source of drinking water is a borehole (HR = 3.03, CI 1.72 to 5.34) or a well (HR = 2.31, 1.20–4.43) have a higher incidence of death compared to those supplied by piped water. Children born to HIV-positive mothers (HR = 4.22, CI 2.68 to 6.65) are approximately four times more likely to die compared to those whose mothers are HIV negative. More children died in the period 2000–2005 compared to those born during 2006–2014 (HR = 9.13, CI 5.70 to 14.6).

**Table 4:** Hazard ratios for potential determinants of under 5 mortality in rural KwaZulu Natal (South Africa) using a Cox proportional hazards regression model.

		Univariable			Multivariable					
Explanatory variables	Categories of explanatory variables	Hazard ratio	95% Confidence Interval	p- value	Adjusted Hazard Ratio	95% Confidence interval	p-value	Relative risk	PAF (%)	95% CI
**Wealth Index**	Poor	3.29	2.126–5.10	<0.001	2.47	1.56–3.93	<0.001	2.45	38.9	22–53.9
	Rich	1			1					
**Source of drinking water**	Borehole	4.844	3.132–7.49	< .0001	3.03	1.72–5.34	<0.001	2.68	9.7	4.2–17.3
	Well	2.951	1.679–5.19	0.0002	2.31	1.20–4.43	0.012	1.65	3.2	-0.2–8.6
	Surface/rain/lake	1.863	1.171–2.97	0.0086	1.07	0.57–1.97	0.840	1.11	1.5	-4.7–10.7
	Other (water tank)	0.310	0.113–0.85	0.0231	0.49	0.18–1.36	0.169	0.59		
	Piped water	1			1					
**Mother’s education**	none	1.643	0.945–2.86	0.0785	0.39	0.05–2.83	0.350	0.83		-1.5–3.9
	Primary	1.390	1.109–1.74	0.0043	0.86	0.50–1.47	0.571	1.11	1.7	-5.0–10.7
	Secondary	1			1					
**Mother’s Vital Status**	Dead	1.811	1.554–2.23	< .0001	3.08	0.40–23.7	0.279	2.23	0.5	-0.3–5
	Alive	1			1					
**Mother’s HIV Status**	Positive	1.675	1.089–1.99	0.0592	4.22	2.68–6.65	<0.001	3.41	18.8	11.2–27.9
	Negative	1								
**Death Period**	2000–2005	7.210	6.014–8.64	<0.001	9.13	5.70–14.6	<0.001	8.48	67.4	57–75.9
	2006–2014	1			1					
**Mother’s age (years) at birth**	< 20	2.673	1.714–4.168	0.301						
	20–29	1.310	0.874–1.963	0.191						
	30–39	1.147	0.754–1.745	0.523						
	≥ 40	1								
**Distance to the national road (km)**	<10	0.875	0.726–1.054	0.160						
	≥10									
**Birth order**	First	0.872	0.748–1.02	0.0796						
	2^nd^, 3^rd^ or 4th	1								
	Fifth or bigger	1.210	0.951–1.54	0.1208						
**Child’s Sex**	Male	1.068	0.812–1.08	0.3679						
	Female	1								
**Distance to the nearest clinic (km)**		1.350	0.699–2.61	0.3712						

### Population Attributable Fractions (PAFs)

Using the multivariable adjusted RR and prevalence of exposure (see methods for formula) we estimated population attributable fractions of 25.4 per cent for wealth index, 25.1 per cent for source of drinking water, 9.3 per cent for mother being HIV-positive, 9.5 per cent for distance to the national road, 18.1 per cent for birth order and 10.6 per cent for time period (2000–2005 v. 2006–2014) in infants. Among children, population attributable fractions of 38.9 per cent for wealth index, 14.4 per cent for source of drinking water, 1.7 per cent for mother’s education, 0.5 per cent for mother’s vital statistics, 18.8 per cent for mother’s HIV-positive, and 67.4 per cent for time period (2000–2005 v. 2006–2014) were estimated. The summed population attributable fraction of community, household and individual risk factors of child mortality for the two age ranges are 102.9 per cent for infant mortality (Table 3) and 141.7 per cent for under-five mortality (Table 4). In addition, child mortality is caused by multiple factors, resulting in the population attributable fraction for individual risk factors overlapping and adding to a percentage of more than 100.

## Discussion

The study’s results show that the average infant and under-five (but not neonatal) mortality from 2006–2014 was significantly lower than that from 2000–2005, which is consistent with a comprehensive pattern in most sub-Saharan countries [31, 8] in recent years. The study also found that socioeconomic status, source of drinking water, mother’s HIV status and period of death are significant risk factors associated with under-five and infant mortality.

The study also demonstrates a clear decreasing trend for under-five mortality during the 2000–2005 period compared to post-2005. This is likely in part due to the rollout of the antiretroviral therapy in 2004, which led to a decrease of HIV related infections and mortality [32]. New HIV infections declined amongst South African children from 3.6 per cent in 2011 to 1.3 per cent in 2017 due to the achievements of the prevention of mother to child transmissions programme [33]. Research has indicated that the decrease in child mortality has largely been influenced by the expansive HIV therapy [34]. South Africa has the biggest antiretroviral treatment programme globally, when the government invested more than US$ 1.34 billion per year to manage the HIV epidemic in 2015 [35], the benefits include increasing life expectancy from 61.2 years in 2010 to 67.7 years in 2015 [36].

The association between mortality risk and HIV-positive mothers is similar to studies in Tanzania, Malawi and Uganda, which found that children born from HIV-infected mothers were more likely to succumb to death compared to those born from HIV-uninfected mothers [37]. This is probably due to the transmission of the HIV virus from mother to child, the disruption of breastfeeding, and the inability of the mother to care for her children when she herself is subject to a terminal illness [23]. The widespread distribution of ART could reduce the effect of mothers being HIV-positive on child mortality through a reduction in transmission, terminal illness and death. This would result in the population attributable fraction of 18 per cent being reduced in relation to the proportion of women who receive ART [38].

Furthermore, HIV infection is a considerable factor in under-five mortality, either directly due to mother-to-child transmission, or indirectly due to maternal inferior health [38]. The death of a mother has adverse effects on the survival of the child, even without the child being infected with HIV, with uninfected children born from HIV positive mothers having a high probability of being orphaned, and being more susceptible to child mortality than those who grow under their own mother’s care [39]. This is largely because of the increased vulnerability to mortality for children born to infected mothers compared to HIV-negative mothers [40]. The death of a mother in many SSA communities potentially results in exploitation of the children by some communities, a lack of support and affection, poverty or even playing a parental role at a young age [41].

Various reasons could account for the lower mortality rates in the period 2006–2014 as compared to 2000–2005, with research showing that it has been extensively attributed to immunisation rates, social grants, improvements in nutrition and the extensive rollout of the antiretroviral therapy programme [3]. Immunisation rates increased from 67 per cent in 2001 to 89.8 per cent in 2014, whilst social grant beneficiaries increased from nine per cent in 2001 to 30 per cent in 2015, and malnutrition decreased from 12.5 to 4.5 per 1,000 children from 2001 to 2015 [3]. Despite the encouraging figures on under-five mortality, South Africa failed to meet the Millennium Developmental Goal 4 in 2015 by two thirds. However, the trend gives some hope towards attaining the 2030 SDG target of an U5MR of less than or equal to 25 deaths per 1,000 live births. It is also important to highlight the insubstantial decline in neonatal mortality as a major challenge globally. This is largely because the neonatal period is the most vulnerable period of a child’s survival. This suggests that in as much as child health interventions like immunisations have been effective in reducing child mortality in the postnatal period, there is still a need to accelerate and scale up interventions like breastfeeding, perinatal period care and others, which have been proven effective in preventing deaths in the neonatal period.

The study also investigated the potential risk factors for under-five mortality across the two age ranges in a South African rural setting. Household wealth was one of the significant factors of under-five mortality in this study, where the risk of mortality was larger for children born into poor versus rich households. This could be explained by higher levels of malnutrition in the poor homesteads, as well as an increased exposure to infections and the inaccessibility of healthcare services [1]. Research has shown that under-five mortality is considerably higher in more deprived households and lower socio-economic groups in developing countries [42].

In addition, the source of drinking water was also found to be an influential factor on under-five mortality, with households using water from wells and boreholes being at a higher mortality risk compared to those using the piped water system. Most water sources in rural sub-Saharan communities are open water bodies, which are easily contaminated by animal and human feces that expose children to diseases related to water, sanitation and hygiene, such as diarrhea, skin conditions, schistosomiasis and trachoma [43].

The study’s findings such as greater under-five mortality risks for children living in households with a poor wealth index are in line with those reported from other studies done in rural SSA [14]. Socio-economic status has been found to have a considerable influence on under-five mortality in rural South Africa [14], in other areas of SSA [44] and in rural Bangladesh. Children born from poor households are more likely to die compared to those from richer households [45]. The significance of the wealth index of a household highlights the importance of the socio-economic status in determining under-five mortality. This could be attributed to the positive impact that a higher socio-economic status has on child health and nutritional status [46]. In South Africa, more than ten per cent of the population live under the optimal poverty line of $1.25 per day [47], which acts as an obstacle for many mothers in accessing adequate healthcare services for their children, leading to high child and infant mortality rates.

Similarly, research has also indicated that there is a higher mortality risk among children aged less than five years old who have access to well water for drinking purposes than those using piped water. Drinking water from unprotected water sources (rivers and dams) has been found to be one of the significant risk factors and determinants of under-five mortality in rural South Africa due to inadequate sanitation and poor hygiene practices [48, 49]. This could be explained by the fact that clean drinking water is one of the most important attributes of a healthier life, particularly in children, whose immune systems are vulnerable to the health hazards posed by bacterial infections, viruses, parasites (schistosomiasis, soil-transmitted helminths, protozoa) and chemicals, in unclean water [50, 51].

As already highlighted in the introduction, most studies have focused on the effect of individual level factors on child mortality, and there remains a gap in the impact of household factors (e.g. wealth index and source of drinking water) [52]. This study has highlighted how the above-mentioned household factors influence child mortality, and the importance of community-based interventions that specifically deal with the survival of children residing in rural areas with low socio-economic statuses, especially in SSA. Policy and health interventions for providing potable water can help eradicate parasitic diseases such as diarrhoea. In addition, educational programmes that focus on hygiene and sanitation can encourage the use of safe water for daily activities, and thereby reduce morbidity. Female education should be encouraged, particularly in rural areas, where decision-making is still dominated by males. Education will empower mothers to make informed decisions about using health facilities for hazards posing a threat to the survival of their children. HIV still needs to be addressed by managing women with HIV infection and should continue to include programmes such as the prevention of mother to child transmission.

Studies in SSA have highlighted the role of mothers’ education on under-five mortality [44, 53]. Knowledgeable mothers are more likely to make well-informed decisions about accessing healthcare services, and maternal healthcare-seeking behaviours [54, 55], such as vaccinations, which could improve child survival. In addition, well-informed mothers are more likely to reside in more economically developed areas with well-equipped medical facilities and sanitary conditions, which will enhance the survival potential of their children [50]. Thus, it is important to develop policies that address the above-mentioned factors to improve the survival potential of the children, specifically those in rural, under-serviced areas.

## Conclusion

This study suggests that under-five mortality in this rural setting has declined from 2000–2014. The findings show that residing in households with a poor socio-economic status, drinking water from unprotected water bodies, having mothers who are HIV-positive, and being born to uneducated mothers are significant risk factors for mortality across both age groups (infant, and under-five) in rural South Africa. There is cause for hope in being able to achieve the SDG 2030 targets in South Africa, for reducing child mortality, which requires good nutrition, safe water, adequate sanitation, good hygiene, and reduced HIV infections. However, much needs to be done to further reduce mortality in these vulnerable age groups. The results highlight the need for a more comprehensive approach to child health intervention programmes towards reducing child mortality in rural SSA. Such policies need to include improvements in socio-economic status, health services, educating mothers, accessibility to clean water sources as well as improving the control of HIV and other preventable diseases. In addition, intervention policies need to embrace comprehensive child feeding guidelines and communication strategies in order to reduce under-five mortality, especially HIV-related deaths.

## Abbreviations

ACDIS: Africa Centre Demographic Information System
ART: Antiretroviral therapy
CI: Confidence Interval
DSA: Demographic Surveillance Area
HR: Hazard Ratio
MDG: Millennium Development Goal
PAF: Population Attributable Fraction
PMTCT: Prevention of mother to child transmission
RR: Relative Risk
SA: South Africa
SSA: Sub-Saharan Africa
SDG: Sustainable Development Goal
U5MR: under-5 mortality rate
VIF: Variance Inflation Factors
