# Correction: Investigating risk factors for under-five mortality in an HIV hyper-endemic area of rural South Africa, from 2000–2014

**DOI:** 10.1371/journal.pone.0306379

**Published:** 2024-06-27

**Authors:** B. Tlou, B. Sartorius, F. Tanser

There are errors in the Author Contributions. The correct contributions are:

**Conceptualization:** B. Tlou

**Data curation:** F. Tanser

**Formal analysis:** B. Tlou

**Funding acquisition:** F. Tanser

**Methodology:** B. Tlou

**Resources:** F. Tanser

**Supervision:** B. Sartorius, F. Tanser

**Writing–original draft:** B. Tlou

After publication of this article [1], the corresponding author notified the journal office that there are errors in the data analysis underlying their paper. The corresponding author explained that for the survival analysis, person time used in the original analyses consisted of the last observed episode for each child rather than the total person time contributed from birth or immigration to censoring, death or out-migration. This leads to underestimation of person time at risk, which impacts both the rate calculations and, to a lesser degree, the risk factor analysis. While the overall secular trend over the period remains largely unchanged, this error also leads to overestimation of the mortality rates concerned. The corresponding author reran the models using the fuller person time offsets for each child and notes that the effect size for the hazard ratios and significance thereof is largely unchanged.

In addition, the corresponding author clarified that he performed the re-analyses using a complete case analysis (i.e., The included records which had no missing covariate information). This resulted in utilizing 759 deaths for which there was complete data for all covariates/risk factors considered. While a “complete” cohort approach for risk factor analysis may not be a major limitation, in terms of performing a full due diligence, the corresponding author would like to clarify that he should have included details of the full cohort and description of the missing data in the original paper.

The corresponding author provides updates to the Abstract, Results, Tables 2–4 and Fig 2 to correct these errors. Please see the location of the error, the original text, and the author-corrected text here.

**Fig 2:**
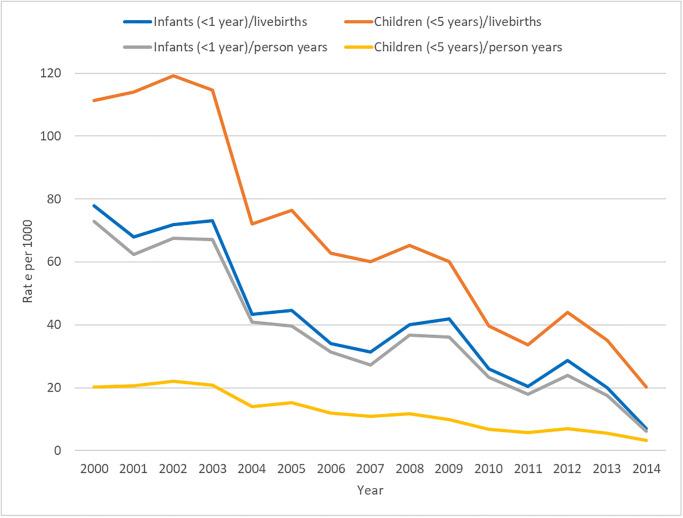
Infant and child deaths per 1,000 live births and person-years between 2000 and 2014.

**Table:** 

Location	Original text	Corrected text
Abstract, Methods subsection, first sentence	We conducted a statistical analysis of 759 births from a population-based cohort in rural KwaZulu-Natal Province, South Africa, from 2000 to 2014.	We conducted a statistical analysis of 759 births deaths with complete covariate/risk factor data from a population-based cohort in rural KwaZulu-Natal Province, South Africa, from 2000 to 2014.
Abstract, Results subsection	Child mortality rates declined by 80 per cent from 2000 to 2014, from >140 per 1,000 persons in years 2001–2003 to 20 per 1,000 persons in the year 2014. The highest under-five mortality rate was recorded in 2002/2003, which decreased following the start of antiretroviral therapy rollout in 2003/4. The results indicated that under-five and infant mortality are significantly associated with a low wealth index of 1.49 (1.007–2.48) for under-fives and 3.03 (1.72–5.34) for infants. Children and infants with a lower wealth index had a significantly increased risk of mortality as compared to those with a high wealth index. Other significant factors included: source of household drinking water (borehole) 3.03 (1.72–5.34) for under-fives and 2.98 (1.62–5.49) for infants; having an HIV positive mother 4.22 (2.68–6.65) for under-fives and 3.26 (1.93–5.51) for infants, and period of death 9.13 (5.70–14.6) for under-fives and 1.28 (0.75–2.20) for infants. Wealth index had the largest population attributable fraction of 25.4 per cent.	Child mortality rates declined by ~81 per cent from 2000 to 2014, from 111.2 deaths per 1,000 livebirths in 2000 to 20.3 per 1,000 livebirths in 2014. The highest under-five mortality rates were recorded in 2001–2003, which decreased following the start of antiretroviral therapy rollout in 2003/4. The multivariable hazard results indicated that infant mortality was significantly associated with a low wealth index with a hazard ratio of 1.67 (1.43–1.97) for infants residing in the poorest households. While this was significant risk factor for children under 5 in the bivariate analysis, poverty was no longer associated with child mortality after multivariable adjustment Other significant factors included: source of household drinking water (borehole) [HR 2.50 (1.68–3.72) for under-fives and 3.12 (2.22–4.38) for infants respectively]; having an HIV positive mother [HR 2.23 (1.66–2.98) for under-fives and 2.80 (2.17–3.62) for infants respectively], and earlier period [2000–2005] [HR 1.99 (1.46–1.54) for under-fives and 1.72 (1.3–2.28) for infants respectively]. Mother HIV positivity and poverty were the two most modifiable population attributable risk factors for infant mortality at 15.4 and 14.7% respectively. Among all children under 5 mother HIV positivity and other water source were the most attributable risk factors respectively at 11.1 and 8.4% respectively.
Results, Description of the Study Population subsection, first paragraph	Table 1 presents the individual, household and community level characteristics of children by mortality status. A total of 759 deaths of children below the age of five years occurred between 2000 and 2014 from a total of 12,989 live births: 274 deaths between 0 and 28 days (neonatal mortality); 277 between one month and 11 months (infant mortality); 208 in the child mortality phase (12–59 months), with the overall U5MR (scaled by live births) being 58.4 deaths per 1,000 live births.	Table 1 presents the individual, household and community level characteristics of children by mortality status. A total of 1705 deaths of children below the age of five years occurred between 2000 and 2014 from a total of 32,537 live births: 169 deaths between 0 and 28 days (neonatal mortality); 872 between one month and 11 months (infant mortality); 664 in the post infancy phase (12–59 months), with an overall period U5MR (scaled by live births) of 27 deaths per 1,000 live births. The infant mortality rate per 1000 livebirths for the period was 42.3 per 1000.
Results, Description of the Study Population subsection, second paragraph	Approximately 70 per cent of the neonatal, infant, child and under-five deaths occurred between 2000 and 2005, with households in the poor quintile having a higher percentage of deaths than wealthier ones (57.1% neonatal, 50.6% infant, and 52.2% under-5). The frequency of male deaths was slightly higher than that of female deaths but this difference was not statistically significant (52.1% vs 47.9%; p = 0.484).	Approximately 70 per cent of the neonatal and 60 percent of infant, child and under-five deaths occurred between 2000 and 2005, with households in the poorest quintile having a higher percentage of deaths than children residing in wealthiest households (57.1% neonatal, 50.6% infant, and 52.2% under-5). The frequency of male deaths was slightly higher than that of female deaths but this difference was not statistically significant (52.1% vs 47.9%; p = 0.484).
Results, Description of the Study Population subsection, third paragraph	Mortality rates per 1,000 live births declined in all child age groups from 2000 to 2014. (Table 2). Between 2000–2005 and 2006–2013, the neonatal mortality rate decreased by approximately six per cent, from 767 deaths per 1,000 person years among live born children (2000–2005) to 722 deaths in 2006–2014 even though the decrease was not statistically significant; infant mortality fell from approximately 50 to 20 deaths; post-neonatal mortality declined by 38 per cent from 379 to 234 deaths; the child mortality rate reduced by approximately 40 per cent, from 33 to 20, and the U5MR dropped by 85 per cent, from 149 to 23 deaths, as shown in Fig 2.	Mortality rates per 1,000 live births declined in all child age groups from 2000 to 2014. (Table 2). Between 2000–2005 and 2006–2013, the neonatal mortality rate decreased by approximately seventy eight per cent (p<0.001), from 11.6 deaths per 1,000 person-years among live born children (2000–2005) to 2.6 deaths per 1,000 person-years in 2006–2014; infant mortality fell from approximately 58 to 25 deaths per 1000; post-neonatal mortality declined by ~50 per cent from 46.4 to 22.3 deaths per 1000; the 1–4 year age band child mortality rate reduced by over 50 per cent, from 9.0 to 4.1 per 1000, and the overall U5MR also dropped by over 50 per cent, from 18.9 to 8.1 deaths, as shown in Table 2 and Fig 2.

The reduction from 2000–2005 to 2006–2014 is more significant, albeit that the rate levels have come down due to the denominator issue alluded to already. Please see the corrected Table 2.

**Table 2:** Trends in early childhood mortality rates in deaths per 1,000-person years among live born children.

Characteristic	2000–2005	2006–2014	p-value
**Neonatal mortality (*N*)**	11.6	2.6	<0.001
**Post neonatal mortality (*PN*)**	46.4	22.3	<0.001
**Infant mortality (1*q*0)**	58.1	24.9	<0.001
**Child mortality (4*q*1)**	9.0	4.1	<0.001
**Under 5 mortality (5*q*0)**	18.9	8.1	<0.001

Neonatal mortality (*N*)-refers to the probability of demising within the first month of life

Post neonatal mortality (*PN*)-refers to the difference between neonatal and infant mortality

Infant mortality (1*qo*)-refers to the probability of dying in the first year of life

Child mortality (4*q*1)-refers to the probability of demising between exact age one and five

Under 5 mortality (5*q*0)-refers to the probability of demising between birth and exact age five.

Fig 2 needs to be updated to reflect the change in the y-axis values, rather than any changes to trend pattern over the period. The authors clarify that secular trend pattern remains the same but the rate value changes due to the use of the incorrect denominator (last episode only rather than full person time contribution under 5 years of age and for relevant age bands within the <5 range). Please see the corrected Fig 2.

After reanalysis, the major risk factors following multivariable adjustment remain the same with very minor changes in coefficient values and associated population attributable fraction estimates, namely: poverty (residing in a household in the poorest socio-economic quantile), non-piped water source, mother HIV status, mother vital status and period of death (2000–2005). In addition, mother vital status was not included in the original multivariable infant model and should have been based on its bivariate association. It is included in the corrected multivariable model and is a significant determinant for infant mortality in Tables 3 and 4. Please see the corrected Tables 3 and 4

**Table 3:** Hazard ratios for potential determinants of infant mortality in rural KwaZulu Natal (South Africa) using a Cox proportional hazards regression model.

		Univariable			Multivariable					
Explanatory variables	Categories of explanatory variables	Hazard ratio	95% CI	p- value	Adjusted Hazard Ratio	95% CI	p-value	Pe	PAF (%)	95% CI
**Wealth Index**	Poorest	2.87	1.76–3.24	<0.001	1.87	1.12–3.1	<0.001	19.86	14.7	2–29
	Wealthiest	1			1					
**Source of drinking water**	Borehole	4.28	3.2–5.72	<0.001	3.12	2.22–4.38	<0.001	5.45	10.4	6–16
	Well	2.93	2.01–4.27	<0.001	1.90	1.2–3.01	0.006	4.1	3.6	1–8
	Surface/rain/lake	1.97	1.5–2.58	<0.001	1.27	0.92–1.75	0.152	13.95	3.6	-1–9
	Other (water tank)	0.40	0.23–0.7	0.001	0.48	0.28–0.83	0.009	13	-7.2	-10-2
	Piped water	1			1					
**Mother’s HIV Status**	Positive	1.69	1.44–1.99	<0.001	2.80	2.17–3.62	<0.001	10.14	15.4	11–21.
	Negative	1			1					
**Distance to national road(km)**	≥10	1.08	0.9–1.31	0.406						
	< 10	1								
**Birth order**	First	0.93	0.82–1.04	0.202						
	2^nd^, 3^rd^ or 4th	1								
	Fifth or bigger	0.94	0.72–1.24	0.676						
**Period of death**	2000–2005	2.16	1.92–2.23	<0.001	1.72	1.3–2.28	<0.001	41.57	23.1	11–35
	2006–2014	1			1					
**Maternal education**	None	1.55	1–2.39	0.05	1.15	0.54–2.46	0.716	1.61		
	Primary	1.47	1.24–1.75	<0.001	1.42	1.05–1.91	0.023	12.64	5	1–10
	Secondary	1			1					
**Mother’s Vital Status**	Dead	5.45	4.66–6.36	<0.001	3.72	2.56–5.39	<0.001	3.54	8.8	5–13
	Alive	1								
**Mother’s age(years) at birth**	< 20	0.89	0.74–1.07	0.207						
	20–29	1.15	1–1.34	0.057						
	30–39	1								
	≥ 40	0.83	0.55–1.26	0.379						
**Child’s Sex**	Male	1.09	0.97–1.23	0.132						
	Female	1								
**Distance to the nearest clinic(km)**	≥10	0.79	0.41–1.52	0.482						
	<10	1								

Pe:Prevalence of exposure

CI: Confidence Interval

**Table 4:** Hazard ratios for potential determinants of under 5 mortality in rural KwaZulu Natal (South Africa) using a Cox proportional hazards regression model.

Univariate					Multivariable				
Explanatory Variables	Categories of Explanatory Variables	Hazard Ratio	95% Confidence Interval	p-value	Adjusted Hazard Ratio	95% Confidence Interval	p-value	PAF (%)	95% Confidence Interval
**Wealth Index**	Poorest	2.02	1.49–2.74	<0.001	1.19	0.77–1.85	0.434	3.7	-5 to 14%
	Wealth	1 (ref)			1 (ref)				
**Source of drinking water**	Borehole	3.86	2.85–5.83	<0.001	2.50	1.68–3.72	<0.001	7.2	3 to 12%
	Well	3.44	2.43–4.87	<0.001	2.28	1.42–3.64	0.001	5.1	2 to 10%
	Surface rain/lake	1.28	0.93–1.76	0.128	0.89	0.59–1.33	0.565	-1.6	-6 to 4%
	Other (Water tank)	0.38	0.22–0.66	0.001	0.42	0.23–0.76	0.004	-8.4	-11 to—3%
	Piped Water	1 (ref)			1 (ref)				
**Maternal Education**	None	1.10	0.65–1.87	0.727	1.38	0.7–2.72	0.349	0.9	-1 to 4%
	Primary	1.54	1.26–1.89	<0.001	1.08	0.76–1.54	0.67	1.1	-3 to 7%
	Secondary	1 (ref)			1 (ref)				
**Mother’s Vital Status**	Dead	5.25	4.34–6.35	<0.001	3.95	1.93–5.26	<0.001	6.5	3 to 12%
	Alive	1 (ref)			1 (ref)				
**Mother’s HIV Status**	Positive	1.69	1.44–1.99	<0.001	2.23	1.66–2.98	<0.001	11.1	6 to 17%
	Negative	1 (ref)							
**Period of Death**	2000–2005	2.25	1.96–2.58	<0.001	1.99	1.46–1.54	<0.001	29.2	16 to 42%
	2006–2014	1 (ref)			1 (ref)				
**Mother’s Age(years) at birth**	<20	0.97	0.78–1.21	0.799					
	20–29	1.10	0.92–1.31	0.302					
	30–39	1 (ref)							
	≥40	0.76	0.46–1.25	0.275					
**Distance to National Road (km)**	≥10	1.06	0.84–1.35	0.606					
	<10	1 (ref)							
**Birth Order**	1^st^	0.93	0.81–1.08	0.346					
	2^nd^, 3^rd^ or 4^th^	1 (ref)							
	≥5^th^	1.09	0.82–1.45	0.537					
**Child’s Sex**	Male	1.07	0.93–1.22	0.345					
	Female	1 (ref)							
**Distance to the nearest(km)**	≥10	0.79	0.41–1.52	0.482					
	<10	1 (ref)							

It is highlighted in the Discussion that socioeconomic status, source of drinking water, mother’s HIV status, and period of death are significant risk factors associated with under-five and infant mortality. The corresponding author would like to clarify that even though the same conclusions still hold based on the corrected findings, socioeconomic status is not statistically significant for under-five mortality following multivariable adjustment for this age band.
